# Cardiovascular Applications of Photon-Counting CT Technology: A Revolutionary New Diagnostic Step

**DOI:** 10.3390/jcdd10090363

**Published:** 2023-08-25

**Authors:** Antonella Meloni, Filippo Cademartiri, Vicenzo Positano, Simona Celi, Sergio Berti, Alberto Clemente, Ludovico La Grutta, Luca Saba, Eduardo Bossone, Carlo Cavaliere, Bruna Punzo, Erica Maffei

**Affiliations:** 1 Department of Radiology, Fondazione G. Monasterio CNR-Regione Toscana, 56124 Pisa, Italy; antonella.meloni@ftgm.it (A.M.); positano@ftgm.it (V.P.); clemente@ftgm.it (A.C.); emaffei@ftgm.it (E.M.); 2 Unità Operativa Complessa di Bioingegneria, Fondazione G. Monasterio CNR-Regione Toscana, 56124 Pisa, Italy; 3 BioCardioLab, Fondazione G. Monasterio CNR-Regione Toscana, 54100 Massa, Italy; simona.celi@ftgm.it; 4 Diagnostic and Interventional Cardiology Department, Fondazione G. Monasterio CNR-Regione Toscana, 54100 Massa, Italy; sergio.berti@ftgm.it; 5 Department of Radiology, University Hospital “P. Giaccone”, 90127 Palermo, Italy; lagruttaludovico@gmail.com; 6 Department of Radiology, University Hospital of Cagliari, 09042 Monserrato, CA, Italy; lucasabamd@gmail.com; 7 Department of Cardiology, Ospedale Cardarelli, 80131 Naples, Italy; ebossone@hotmail.com; 8 Department of Radiology, Istituto di Ricerca e Cura a Carattere Scientifico SynLab-SDN, 80131 Naples, Italy; carlo.cavaliere@synlab.it (C.C.); bruna.punzo@synlab.it (B.P.)

**Keywords:** photon-counting detectors, computed tomography angiography, heart, coronary arteries

## Abstract

Photon-counting computed tomography (PCCT) is an emerging technology that can potentially transform clinical CT imaging. After a brief description of the PCCT technology, this review summarizes its main advantages over conventional CT: improved spatial resolution, improved signal and contrast behavior, reduced electronic noise and artifacts, decreased radiation dose, and multi-energy capability with improved material discrimination. Moreover, by providing an overview of the existing literature, this review highlights how the PCCT benefits have been harnessed to enhance and broaden the diagnostic capabilities of CT for cardiovascular applications, including the detection of coronary artery calcifications, evaluation of coronary plaque extent and composition, evaluation of coronary stents, and assessment of myocardial tissue characteristics and perfusion.

## 1. Introduction

Cardiovascular diseases (CVDs) are the commonest cause of death worldwide and a major contributor to disability and impaired quality of life [1]. The increasing age of the global population and improvements in survival rates are expected to lead to a greater significance of CVDs in the future. Addressing the challenge of CVDs requires a comprehensive approach that includes preventive measures, early detection, effective management, and access to appropriate medical interventions. Non-invasive cardiac imaging plays a vital role in this process, enabling healthcare professionals to diagnose and evaluate the severity of cardiovascular conditions, monitor treatment outcomes, and guide therapeutic decision making. Advances in imaging technology have improved the accuracy and efficiency of these diagnostic procedures, offering valuable insights into the structure and function of the heart and blood vessels.

Cardiovascular computed tomography (CT) has experienced exponential growth in recent years and has become an integral part of clinical practice for evaluating a wide range of cardiovascular conditions [2,3]. The widespread adoption of cardiovascular CT can be attributed to its non-invasiveness and its ability to provide high-resolution images, rapid acquisition times, and three-dimensional reconstructions. However, there is still the critical need to obtain better contrast resolution and image quality at lower radiation doses, reduce blooming or beam-hardening artifacts, and improve tissue characterization capabilities [4,5,6]. Photon-counting CT (PCCT) is a promising technology that holds the potential for further improving cardiovascular CT imaging and achieving the desired goals [7]. PCCT utilizes photon-counting detectors (PCDs), which offer several benefits compared to conventional energy-integrating detectors (EIDs) used in traditional CT scanners [8,9,10,11].

This narrative review will provide an overview of PCCT by describing its technical principles, highlighting its advantages compared to conventional CT technology, and presenting its current applications in cardiovascular imaging.

## 2. Photon-Counting Detector Technology

Conventional EIDs utilize a two-step indirect conversion process. Initially, the incident X-ray photons are transformed into visible light through a scintillator. Subsequently, these secondary photons are absorbed by a photodiode array and converted into electrical impulses. By integrating the energy of all X-ray photons over a specific timeframe, the detector loses the ability to retain the energy information of individual photons [12]. To avoid optical cross-talk, in EIDs, the individual detector cells are divided by optically opaque layers called septa. However, these septa create inactive areas on the detector surface and, due to their limited thickness, they impact the geometric dose efficiency [12,13].

**Figure 1:**
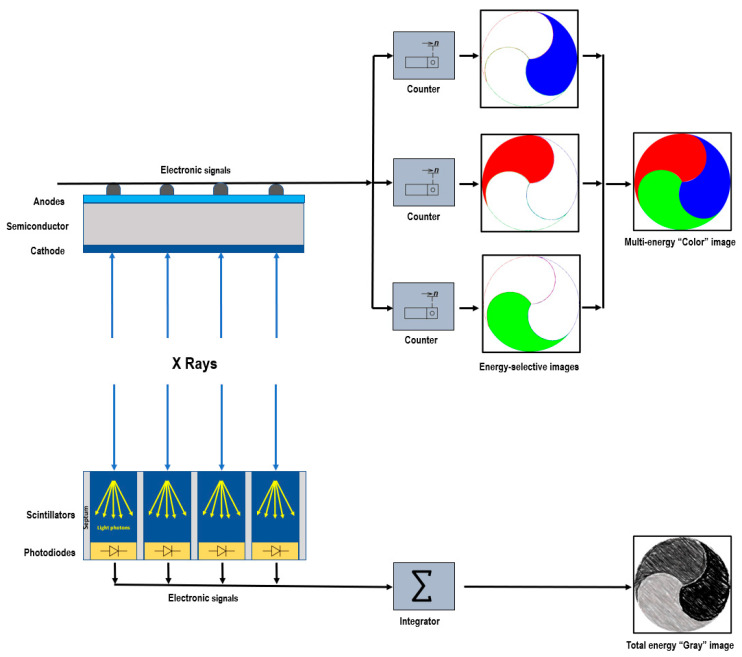
Schematic representation of photon-counting detector directly converting X-rays into an electrical signal (**top**) and of an energy-integrating detector (**bottom**).

PCDs employ a direct conversion technique [14,15]. They consist of a semiconductor layer made of cadmium telluride, cadmium zinc telluride, or silicon, with a large-area cathode electrode on the upper side and pixelated anode electrodes on the lower side (Figure 1). Applying a high voltage, typically between 800 and 1000 V, between the cathode and individual anodes creates a strong electric field. When incident X-rays are absorbed within the semiconductor, charges in the form of electron–hole pairs are generated. These charges, under the influence of the electric field, separate and move toward the anodes. As the electrons reach the anodes, they produce short current pulses, which are converted into voltage pulses by an electronic pulse-shaping circuit [16]. Since the pulse height is proportional to the photon’s energy, PCDs provide energy information for each detected photon through their output signal. The output signal from the PCD is processed by multiple electronic comparators and counters. The count of the number of generated pulses makes it possible to determine the quantity of the interacting X-ray photons.

Furthermore, pulse heights can be compared with a voltage corresponding to a particular photon energy level, denoted as energy threshold [17]. When the energy level of a detected photon is higher than the threshold value, the photon count is incremented by one. This counting process allows us to measure the number of photons with energy equal to or greater than the specified energy level [18] and to sort them into several energy bins (typically two to eight). The lower threshold is typically set higher than the electronic noise level to ensure that the noise is effectively eliminated or suppressed in the final signal. The other thresholds can be uniformly spaced or chosen strategically to optimize the desired imaging outcome [19].

## 3. Benefits of PCDs

This section describes the benefits the PCCT system offers compared to conventional CT technology.

### 3.1. Higher Spatial Resolution

The spatial resolution of a CT measurement is influenced by several factors, including some physical properties of the scanner, like the X-ray source (focal spot size) and the detector (pixel size and scattering).

Recent advancements in EIDs have led to improvements in spatial resolution, with pixel pitches being approximately 0.5 mm at the detector [20]. However, the need for highly reflecting layers poses challenges in manufacturing smaller detector element areas. The septa cannot be made too thin, as it would result in photon cross-talk and degrade image quality. Additionally, the design of smaller detector pixels would cause an overall decrease in the detector area sensitive to X-rays, with a consequent decrease in the geometric dose efficiency [16].

Since in PCDs there are no reflectors or dead areas between pixels, the pixels can be made smaller without sacrificing the geometric efficiency [16]. The pixel pitch can reach 0.15–0.225 mm at the isocenter [21,22,23,24], translating into a higher spatial resolution. The improved spatial resolution is the key to the generation of clearer and more detailed images and to the reduction in partial volume effects.

### 3.2. Increased Contrast

In conventional EIDs, photons are typically weighted based on their energy, and high-energy photons tend to contribute more to the signal than low-energy photons. Since low-energy photons carry valuable information about the contrast between different materials, their underweighting can reduce the contrast-to-noise ratio (CNR) [16,25]. Additionally, the non-uniform weighting of photons leads to an increase in the variance relative to the mean value, reducing the signal-to-noise ratio (SNR). This phenomenon is known as the Swank factor [26].

In PCDs, all photons are equally weighted regardless of their energy (one photon, one count). Assigning relatively more weight to low-energy photons can result in higher contrast than EIDs, especially for materials with low X-ray attenuation [27,28,29,30]. Importantly, the weighting scheme in PCDs can be customized to optimize the CNR for specific materials or imaging tasks [16,31]. Moreover, PCDs, by counting each photon individually, eliminate the Swank factor and its associated impact on image quality.

### 3.3. Noise Reduction

The energy-discriminating ability of PCDs is instrumental in minimizing the impact of the electronic noise, which can decrease image uniformity and cause noticeable streak artifacts. When the low-energy threshold of a PCD is set above the noise-floor level (typically around 20–25 keV), any electronic noise signals below this threshold are not considered valid photon events or pulse counts [18]. However, although the electronic noise can be effectively removed from photon and/or pulse counts, it still persists in the spectral information.

The capacity of PCDs to eliminate electronic noise is particularly advantageous in scenarios involving low-dose CT scans or patients with high body mass, where noise can be a significant concern. In such scenarios, PCDs have been found to provide reduced streak artifacts, improved signal uniformity, and more stable CT numbers than conventional EIDs [32,33]. Importantly, the reduced noise levels in PCDs allow for dose-efficient imaging, achieving comparable image quality to EIDs while using lower radiation doses. These benefits contribute to improved diagnostic accuracy and patient safety in CT imaging.

### 3.4. Multienergy Acquisition

Spectral CT harnesses the intrinsic energy-dependent information embedded within CT images, enabling a deeper understanding of the underlying tissue composition and opening up new avenues for diagnostic imaging.

One of the primary mechanisms employed in spectral CT is material decomposition. The application of sophisticated algorithms on a series of energy-selective images allows to generate a collection of basis image maps. The number of bases aligns with the quantity of gathered spectral information (N bases for N spectral data), and it can be enlarged to N + 1 by imposing mass or volume conservation constraints, albeit with a risk of introducing inaccuracies [34]. Each basis image map depicts the concentration of the equivalent substance voxel by voxel. These basic material images provide a multitude of visualization alternatives. They can be immediately showcased to illustrate the distribution of specific materials, such as contrast agents, within the imaged area. Alternatively, these images can go through additional manipulation to generate virtual monochromatic images (VMI) [35,36,37], virtual non-contrast (VNC) images [38], or material-specific color-overlay images [39]. Conventional CT acquires data in two energy regimes and can accurately isolate a single contrast agent, like iodine, from the background without any underlying assumptions. However, it faces limitations when differentiating two contrast materials with high atomic numbers (high Z). PCDs play a crucial role in overcoming this challenge. Their ability to discriminate photons of different energies through pulse-height analysis enables the acquisition of simultaneous multi-energy data (N ≥ 2) with impeccable spatial and temporal registrations and lacking spectral overlap [40]. The expansion of the number of energy regimens in spectral CT significantly enhances the precision of measuring each photon energy, leading to improved material-specific or weighted images [41,42]. Concentrations of contrast agents, calcium, or other substances can be quantified independently of acquisition parameters, leading to improved quantitative analysis.

Another advantage of employing multiple energy measurements in spectral CT is the ability to quantify elements with K-edges within the diagnostic energy range. Indeed, by acquiring CT data at multiple energy levels, spectral PCCT can effectively measure the X-ray attenuation profiles of different materials, taking advantage of their distinct K-edge energies. This information enables the identification and quantification of various elements with distinct pharmacokinetics within the same biological system. Multi-energy acquisition opens avenues for the use of alternative contrast agents to iodine, such as gold, platinum, silver, ytterbium, and bismuth [43,44,45,46], and for the development of new types of contrast agents, including nanoparticles targeted to specific cells or enzymes [47,48,49,50]. These unique opportunities pave the way for molecular and functional CT imaging as well as simultaneous multi-contrast agent imaging [39,51,52,53], empowering clinicians with advanced tools for precise diagnosis and treatment planning.

### 3.5. Artifact Reduction

Artifacts are commonly observed in clinical CT and can mimic or obscure true pathology.

Beam-hardening artifacts occur when the X-ray beam passing through an object is more attenuated by high-density materials than by low-density materials (soft tissues). This uneven attenuation causes a distortion of the reconstructed CT images, resulting in streaking or shading artifacts [54]. In PCDs, constant weighting allows normalizing the attenuation measurements from different energy levels, reducing the beam-hardening artifacts [55,56]. In this context, using high-energy thresholds that act as a filter is particularly advantageous [32,57].

Calcium-blooming and metal artifacts are caused by volume averaging, motion, and beam hardening [58]. These artifacts are significantly mitigated in PCDs thanks to the improved spatial resolution and the consequent decrease in partial volume effects and thanks to the improved material decomposition allowing for accurate separation of the high-density materials (such as metals) from the surrounding soft tissues [59].

PCCT’s faster acquisition times enable shorter scan durations, reducing the chance of motion artifacts caused by patient movement.

## 4. Challenges of PCCT Technology

Alongside the advantages, there are also limitations associated with using PCCT technology. These limitations must be considered when assessing its potential impact on clinical CT imaging.

### 4.1. Technical Challenges

PCCT faces technical challenges such as charge sharing, pixel cross-talk, and pulse pile-up [13,60].

Charge sharing is a phenomenon that occurs in PCDs when X-ray photons arrive near the boundary between pixels. In such cases, the charge generated by the X-ray interaction can spread or “share” across multiple adjacent pixel electrodes. As a result, the charge may be detected in more than one pixel, leading to the incorrect assignment of charge to multiple pixels instead of just one [61,62,63]. When charge sharing occurs, it can distort the distribution of detected photons and introduce artifacts in the image. Researchers and manufacturers continue exploring and refining techniques (development of advanced correction methods and optimization of detector design) to minimize the effects of charge sharing in PCDs. Besides charge sharing, there are other types of pixel cross-talk that can affect the performance of the detector, like the K-fluorescence escape. This phenomenon occurs when secondary photons from fluorescence in the detector material escape the original pixel and are detected in neighboring pixels [18]. These effects place a lower limit on the practical pixel size in PCD applications.

Pulse pile-up occurs when the generated voltage pulses from individual photon interactions overlap in time, especially at very high X-ray flux rates. This overlapping of pulses can cause errors in photon counting and energy measurement, resulting in distorted energy spectra and compromising image quality [64,65]. One way to mitigate pulse pile-up is to decrease the pixel size of the detector. However, it is important to note that at realistic X-ray flux rates encountered in clinical CT imaging, the effect of pulse pile-up is generally not a major concern [64].

### 4.2. Contrast Agents and K-Edge Imaging

Some clinical applications of PCCT involve using alternative contrast agents [47,50].

Compared to other imaging modalities, PCCT may require higher doses of gadolinium. The higher dosage can raise concerns about patient safety and potential adverse effects. It is crucial to carefully monitor and optimize the dosage to balance diagnostic accuracy and potential risks.

The clinical translation of nanoparticles is still in the experimental stage, and further research is needed to evaluate their safety, efficacy, and potential advantages over conventional contrast agents.

### 4.3. Clinical Validation

While PCCT shows promising potential in cardiovascular imaging and other applications, further clinical validation and comparative studies are needed to establish its diagnostic accuracy, clinical utility, and impact on patient outcomes.

### 4.4. Cost and Availability

The development and production of PCD are expensive, and the high cost poses a significant barrier to the widespread adoption of PCCT in clinical settings [66]. However, when the technology becomes more established, a significant cost reduction is expected to occur.

Currently, the only clinical scanner with PCCT capabilities is the NAEOTOM ALPHA from Siemens (Germany). It was developed on the platform of the most developed EID scanner (non-PCD detector; state-of-the-art Dual-Source CT scanner, FORCE from Siemens), and it has basically the same starting technical features. Therefore, the NAEOTOM ALPHA is a development of the highest performing non-PCCT scanner.

### 4.5. Acquisition of Images in Cardio-Synchronized Exam

There are limits that are related to the scanner features. Anatomical coverage is not limited because it can be achieved with ultra-fast high-pitch (FLASH) protocol (pitch 3.2). The temporal resolution is the same as the best non-PCCT scanner (i.e., 66 ms). Arrhythmias and high heart rate require the same approach as the above-mentioned state-of-the-art Dual-Source non-PCCT scanner (e.g., systolic scan and retrospective ECG gating) [67].

## 5. Cardiovascular Applications of PPCT

The improved diagnostic performance of PCCT over conventional CT in the diagnosis and characterization of cardiovascular diseases has been demonstrated in several phantom, animal, and even human studies. This section provides a summary of these studies.

Table 1 sums up the applications and the main advantages of PCCT in cardiovascular imaging.

### 5.1. Coronary Lumen Detection

Coronary CT angiography (CCTA) has established itself as a reliable and robust first-line imaging modality for evaluating coronary artery stenosis severity [68]. However, the diagnostic performance of conventional CCTA is hampered by the limited spatial resolution and by the presence of severely calcified plaques, which cause blooming artifacts. The blooming artifacts often render the examinations non-diagnostic in patients with significant and disseminated coronary calcifications or overestimate stenosis and false-positive diagnoses, affecting patient care and management [69,70].

Several studies have proven the utility of PCCT in improving the measurements of plaque volume and stenosis severity, attributed to the increase in spatial resolution, the better soft-tissue contrast, and the reduced noise.

Si-Mohamed et al. [59] demonstrated in a phantom study that PCCT images were characterized by a 2.3- and 2.9-fold higher detectability index for coronary lumen and non-calcified plaque, respectively, compared to EID-CT images [59]. In the same study, a clinical validation was also performed, and both PCCT and conventional CT angiography were performed in 14 patients. The results of the image analysis, performed by three radiologists, confirmed an increased image quality and diagnostic confidence for PCCT images [59].

The unique capabilities of PCCT prove particularly advantageous when it comes to evaluating luminal stenosis within heavily calcified plaques. Koons et al. emulated in their phantom study coronary arteries with calcifications of varying shapes and sizes and demonstrated that, when compared to conventional CT at a matched dose, PCCT offered improved visualization of calcium plaques and patent lumen and superior accuracy in the quantification of luminal stenosis across all plaque types [11]. Interestingly, for the heaviest calcification analyzed, which involved a ring-shaped plaque resulting in a 75% area stenosis, only the images generated by PCCT could detect the presence of iodine within the lumen, indicating that the vessel was not fully blocked. In the study by Li et al. [71], a novel method was proposed for determining the percent area of stenosis that relied on the material decomposition of dual-energy and multiple-energy CT images, eliminating the need for segmentation. The authors demonstrated through computer simulations that this approach effectively mitigated partial volume and blooming effects and that accurate and reproducible stenosis measurement could be achieved in phantom experiments by utilizing multiple-energy CT images. Notably, when comparing the performance of four-threshold PCCT images with dual-energy CT (DECT) and two-threshold PCCT images, the four-threshold PCCT images exhibited lower estimation errors, suggesting that this approach was more effective in minimizing measurement inaccuracies. Additionally, the study employed three-basis-material decomposition directly on the four-threshold PCCT images, resulting in the generation of calcium, iodine, and water maps. In the already mentioned in vivo study, Si-Mohamed et al. focused specifically on calcified coronary plaques. They revealed a significant reduction in blooming artifacts with PCCT images compared to EID-CT images [59].

When evaluating vessel lumen, emerging image reconstruction algorithms based on spectral CT introduce a different approach. Allmendinger et al. [72] assessed the performance of a novel calcium-removal image reconstruction algorithm called PureLumen. Their research concentrated on the exclusive removal of the calcified contribution within an anthropomorphic thorax phantom. The algorithm effectively mitigated the negative impact of blooming artifacts, resulting in clearer and more accurate imaging.

**Figure 2:**
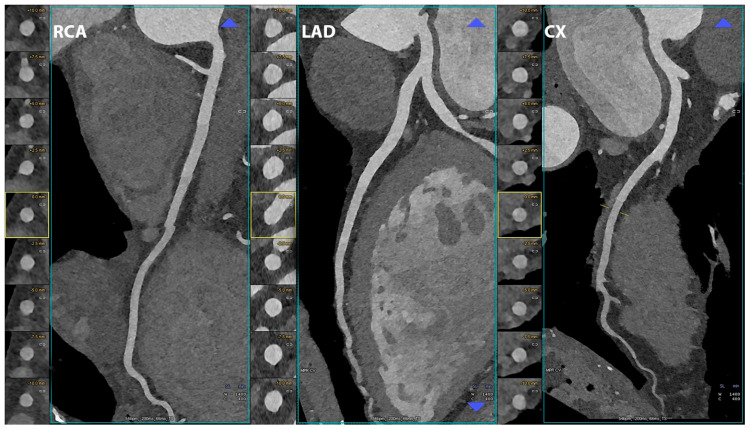
Cardiac/coronary PCCT examples of normal coronary arteries.

**Figure 3:**
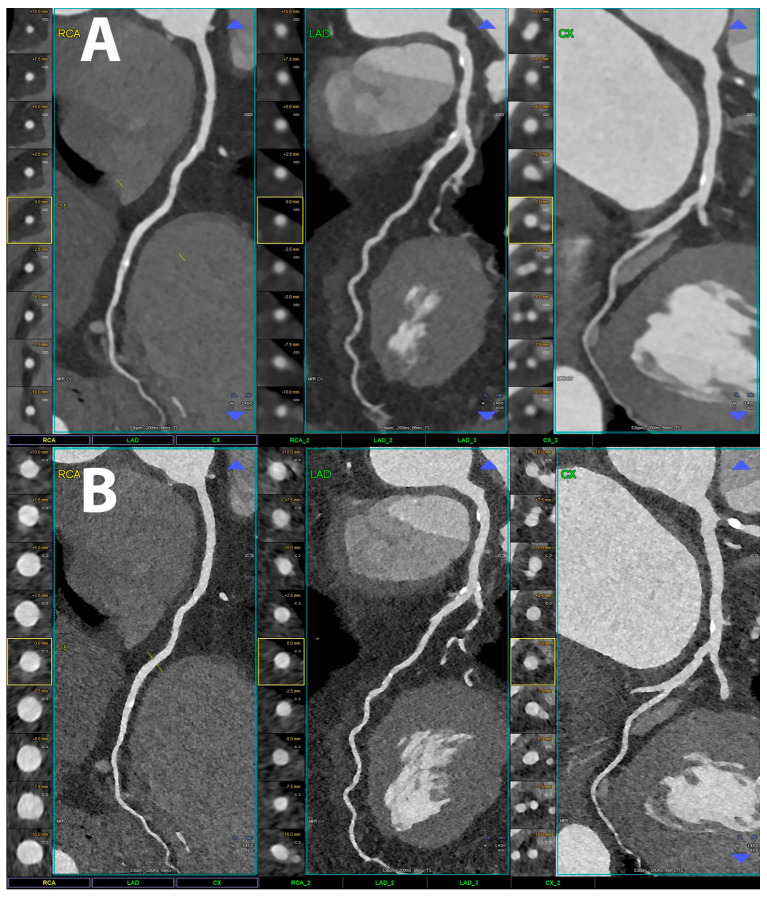
Cardiac/coronary PCCT examples of coronary arteries with standard vs. ultra-high spatial resolution.

**Figure 4:**
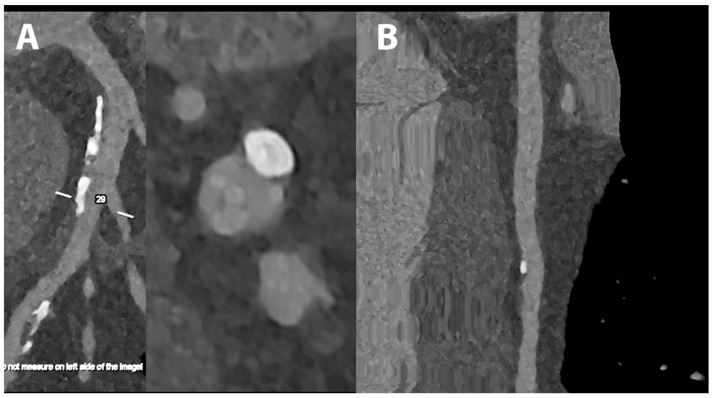
Cardiac/coronary PCCT example of coronary arteries with moderate calcifications.

**Figure 5:**
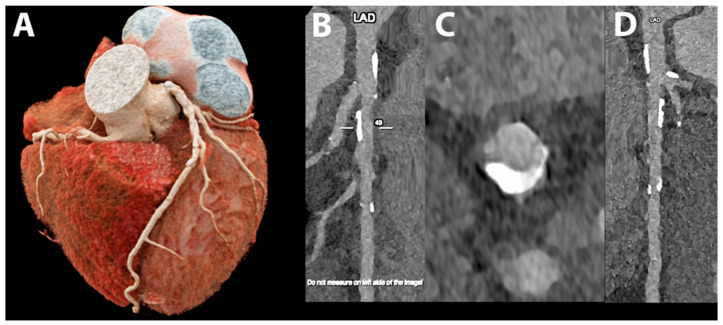
Cardiac/coronary PCCT example of coronary arteries with moderate calcifications.

**Figure 6:**
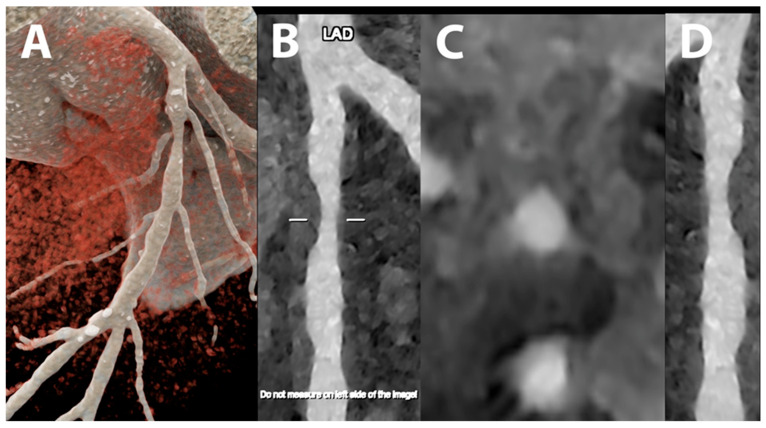
Cardiac/coronary PCCT example of a significant stenosis of proximal left anterior descending coronary artery.

**Figure 7:**
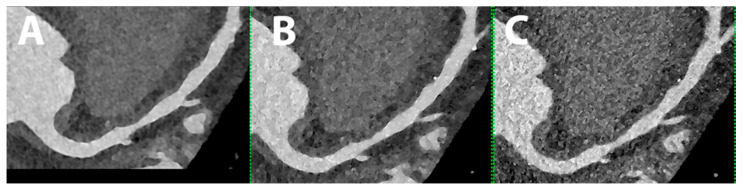
Cardiac/coronary PCCT example of non-significant stenosis of proximal left anterior descending coronary artery.

Examples of coronary PCCT images are shown in Figure 2, Figure 3, Figure 4, Figure 5, Figure 6 and Figure 7.

In the three main panels a curved multiplanar reconstruction along the central lumen line of principal coronary arteries is displayed for the right coronary artery (RCA), the left anterior descending coronary artery (LAD), and for the left circumflex artery (CX); on the left side of each coronary artery there are axial short-axis lumen visualizations. The scan was performed on a commercial whole-body Dual-Source Photon-Counting CT scanner (Naeotom Alpha, Siemens Healthineers, Munich, Germany) with 0.2 mm slice thickness, 0.1 mm reconstruction increment, and FOV 140 mm; the scan was performed with retrospective ECG gating with tube current modulation. In this case, coronary arteries were normal (no calcium and no non-calcified atherosclerosis) and are displayed with a resolution matrix of 1024 × 1024 pixels on the source axial reconstructions with a kernel filtering of Bv60 (vascular kernel medium-sharp) and with maximum intensity of Quantum Iterative Reconstruction (QIR 4). The actual displayed resolution is 0.1 mm (100 microns).

The patient shows non-obstructive coronary artery disease (CAD) along the three major coronary arteries displayed in the three main panels as curved multiplanar reconstruction along the central lumen line (i.e., right coronary artery–RCA; left anterior descending coronary artery–LAD; left circumflex artery–CX); on the left side of each coronary artery, there are axial short-axis lumen visualizations. The upper row (A) is reconstructed using conventional spatial resolution parameters that are today’s state-of-the-art but not using photon-counting capabilities; the lower row (B) is reconstructed using the ultra-high resolution achievable with PCCT. The scan was performed on a commercial whole-body Dual-Source Photon-Counting CT scanner (Naeotom Alpha, Siemens Healthineers) with 0.2 mm slice thickness, 0.1 mm reconstruction increment, and FOV 140 mm; the scan was performed with retrospective ECG gating with tube current modulation. In this case, the coronary arteries show non-obstructive atherosclerosis and are displayed in the upper row with 0.6 mm slice thickness, 0.4 mm reconstruction increment, FOV 140 mm, and resolution matrix of 512 × 512 pixels on the source axial reconstructions with a kernel filtering of Bv40 (vascular kernel medium-smooth) and with maximum intensity of Quantum Iterative Reconstruction (QIR 4); in the lower row, there is 0.2 mm slice thickness, 0.1 mm reconstruction increment, FOV 140 mm, and resolution matrix of 1024 × 1024 pixels on the source axial reconstructions with a kernel filtering of Bv60 (vascular kernel medium-sharp) and with maximum intensity of Quantum Iterative Reconstruction (QIR 4). The actual displayed resolution is 0.25–0.4 mm in the upper row and 0.1 mm (100 microns) in the lower row.

The difference in image quality is quite evident; the normal images seem blurred and out of focus compared to 100 microns UHR reconstructions; calcifications are much sharper and completely confined on the wall of the coronary arteries without any significant blooming into the lumen of the vessel.

The patient shows predominantly calcified, non-obstructive coronary artery disease (CAD) along the proximal left main (LM) and left anterior descending coronary artery (LAD) (A—reference curved longitudinal MPR image and axial cross-section of proximal LAD; B—stretched longitudinal MPR).

The dataset was reconstructed using state-of-the-art photon-counting capabilities with ultra-high resolution achievable with PCCT. The scan was performed on a commercial whole-body Dual-Source Photon-Counting CT scanner (Naeotom Alpha, Siemens Healthineers) with 0.2 mm slice thickness (0.1 mm reconstruction increment and FOV 140 mm); the scan was performed with retrospective ECG gating with tube current modulation. In this case, the coronary arteries show non-obstructive atherosclerosis and are displayed with a resolution matrix of 1024 × 1024 pixels on the source axial reconstructions with a kernel filtering of Bv60 (vascular kernel medium-sharp) and with maximum intensity of Quantum Iterative Reconstruction (QIR 4). The actual displayed resolution is 0.1 mm (100 microns).

The clarity and sharpness of the coronary artery and of the diseased atherosclerotic wall is very high; as in the previous example, the calcifications are much sharper and completely confined on the wall of the coronary arteries without any significant blooming into the lumen of the vessel.

The patient shows predominantly calcified, non-obstructive coronary artery disease (CAD) along the proximal left main (LM) and left anterior descending coronary artery (LAD) (A—cinematic volume rendering of the whole heart in upper frontal view; B—reference stretched longitudinal MPR image; C—axial cross-section of proximal LAD; D—stretched longitudinal MPR with a higher reconstruction kernel).

The dataset was reconstructed using state-of-the-art photon-counting capabilities with ultra-high resolution achievable with PCCT. The scan was performed on a commercial whole-body Dual-Source Photon-Counting CT scanner (Naeotom Alpha, Siemens Healthineers) with 0.2 mm slice thickness (0.1 mm reconstruction increment and FOV 140 mm); the scan was performed with retrospective ECG gating with tube current modulation. In this case, the coronary arteries show non-obstructive atherosclerosis and are displayed with a resolution matrix of 1024 × 1024 pixels on the source axial reconstructions with a kernel filtering of Bv60 (vascular kernel medium-sharp; B–C)/B72 (vascular kernel sharp; D) and with maximum intensity of Quantum Iterative Reconstruction (QIR 4). The actual displayed resolution is 0.1 mm (100 microns).

The clarity and sharpness of the coronary artery and of the diseased atherosclerotic wall is very high; as in the previous example, the calcifications are much sharper and completely confined on the wall of the coronary arteries without any significant blooming into the lumen of the vessel.

The patient shows a significant stenosis of proximal left anterior descending coronary artery (LAD) due to non-calcified coronary artery disease (CAD) (A—cinematic volume rendering of the proximal left coronary artery in upper frontal view; B—reference stretched longitudinal MPR image; C—axial cross-section of proximal LAD; D—stretched longitudinal MPR with a higher reconstruction kernel).

The dataset was reconstructed using state-of-the-art photon-counting capabilities with ultra-high resolution achievable with PCCT. The scan was performed on a commercial whole-body Dual-Source Photon-Counting CT scanner (Naeotom Alpha, Siemens Healthineers) with 0.2 mm slice thickness (0.1 mm reconstruction increment and FOV 140 mm); the scan was performed with retrospective ECG gating with tube current modulation. In this case, the coronary arteries show non-obstructive atherosclerosis and are displayed with a resolution matrix of 1024 × 1024 pixels on the source axial reconstructions with a kernel filtering of Bv60 (vascular kernel medium-sharp; B–C)/B72 (vascular kernel sharp; D) and with maximum intensity of Quantum Iterative Reconstruction (QIR 4). The actual displayed resolution is 0.1 mm (100 microns).

The clarity and sharpness of the stenosis characterized by a non-calcified atherosclerotic wall is very high, allowing a very precise quantification of the actual degree of stenosis both using a diameter technique and a vessel-area technique; the high quality of the image allows to perfectly plan interventional treatment deciding the actual type and size of the stent required for the interventional treatment.

The patient shows a non-significant stenosis of proximal left anterior descending coronary artery (LAD) due to non-calcified coronary artery disease (CAD) (A, B, and C are all MPR images with increasingly sharper reconstruction kernels).

The dataset was reconstructed using state-of-the-art photon-counting capabilities with ultra-high resolution achievable with PCCT. The scan was performed on a commercial whole-body Dual-Source Photon-Counting CT scanner (Naeotom Alpha, Siemens Healthineers) with 0.2 mm slice thickness (0.1 mm reconstruction increment and FOV 140 mm); the scan was performed with retrospective ECG gating with tube current modulation. In this case, the coronary arteries show non-obstructive atherosclerosis and are displayed with a resolution matrix of 1024 × 1024 pixels on the source axial reconstructions with a kernel filtering of Bv60 (vascular kernel medium-sharp; A)/B68 (vascular kernel sharp; B)/B76 (vascular kernel sharp; C) and with maximum intensity of Quantum Iterative Reconstruction (QIR 4). The actual displayed resolution is 0.1 mm (100 microns).

The capability of creating an image that actually reflects the underlying spatial resolution obtained from the photon-counting detector is very much dependent on the reconstruction parameters; the image can gain or lose spatial resolution because of the kernel applied; higher kernels are recommended when preservation of very high spatial resolution is mandatory (as it is for basically any coronary artery investigation).

### 5.2. Coronary Artery Calcium Score

Coronary artery calcium (CAC) quantification using the Agatston methodology [73,74] holds significant value in predicting cardiovascular events, as it provides incremental prognostic information beyond traditional risk factors [75]. Moreover, it has the potential to act as a screening tool for CAD in asymptomatic individuals, thus enabling targeted interventions and preventive measures [76].

When using conventional CT scanners, the accuracy of CAC quantification can be influenced by two main factors: blooming artifacts, which can result in overestimation of the CAC burden and potentially affect the accuracy of risk stratification, and partial volume effects, which can prevent the accurate detection of thin calcifications, leading to potential underestimation of the CAC burden. The use of PCDs in CT imaging has shown promise in reducing blooming artifacts and improving the accuracy of calcium quantification.

A phantom study demonstrated that, compared to conventional CT, PCCT provided improved CAC detectability and a more precise and accurate determination of volume scores at reduced slice thickness [77]. The main finding of the study was that using monoenergetic (monoE) reconstructions at 70 kiloelectron volts (keV) for PCCT acquisitions at different tube potentials enabled reproducible Agatston scores for medium- and high-density CAC. These findings indicate that PCCT can provide consistent and accurate CAC assessment using specific reconstruction settings. An ex vivo study of cadaveric hearts demonstrated an excellent correlation and agreement between the Agatston scores derived from conventional CT and PCCT. This indicates a good potential for converting the established Agatston score from EID-CT to PCCT technology [9]. Furthermore, the study demonstrated good inter-scan reproducibility for both PCD-CT and EID-CT. Eberhard et al. demonstrated in their phantom and in vivo study that PCCT was capable of accurately quantifying CAC burden and, most importantly, that a more accurate and reliable CAC scoring could be achieved by optimizing the iterative image reconstruction algorithm and increasing the kiloelectron volt levels of the virtual monoenergetic images [78].

When considering the implementation of CAC screening in asymptomatic individuals, it is essential to weigh the potential benefits against the risks associated with ionizing radiation exposure from repeated CT scans. The group of van der Werf demonstrated in several phantom studies that PCCT could maintain high sensitivity for detecting CAC while minimizing radiation dose, particularly when using optimized imaging techniques and energy levels. In comparison with conventional CT, PCCT was effective in maintaining and improving the detection of CAC even at a reduced radiation dose of 50% and allowed for more accurate measurement of physical volumes, especially at reduced slice thickness and for high-density coronary artery calcium [79]. Using monoE reconstructions at 70 keV for PCCT acquisitions at 90 kVp allowed for reproducible Agatston scores for medium- and high-density CAC densities with a decreased radiation dose of up to 67% [80]. In another study, different monoE-level-specific Agatston score thresholds for CAC scoring on PCCT were applied [81]. At reduced monoE levels, there was an increase in CNR for each CAC density, and at a 50% decreased radiation dose, there were negligible differences in Agatston score deviation compared to the reference for specific energy levels. For low-density CAC, this was observed for the energy range of 60 keV, while for medium-density CAC, it encompassed the range of 60 to 120 keV. In the case of high-density CAC, the non-relevant differences in Agatston score deviation were obtained within the range of 40 to 130 keV. Mergen et al. proved in their phantom study, where extension rings were employed to simulate varying patient dimensions, that tube-voltage-independent calcium scoring was also possible with 100 kV and tin filtration [82]. Imaging at 90 kV or Sn100 kV exhibited a size-related decrease in radiation dosage ranging from 23% to 48% when compared to imaging at the standard 120 kV.

A human study confirmed the ability of PCCT to enhance the image quality of CAC scoring and/or reduce radiation dose while preserving diagnostic image quality [83]. For the ex-vivo human hearts (N = 10), the reproducibility of CAC scoring at the lowest dose setting of 50 mAs was found to be significantly higher for PCD compared to the EID scans. Regarding the in vivo scans of 10 healthy volunteers, the agreement between standard-dose and low-dose CAC scores was notably better for the PCD system than the EID system.

Using spectral information, PCCT can reconstruct images without relying on the signal from iodinated contrast agents. This means that PCCT has the potential to eliminate the need for a separate non-enhanced scan and, therefore, to streamline the imaging process and reduce the overall radiation dose to the patient. On a clinical first-generation PCCT system, Emrich et al. compared the accuracy of CAC scoring using a novel virtual noniodine reconstruction technique (PureCalcium) with VNC reconstructions and true non-contrast (TNC) acquisitions [84]. In the phantom study, a strong agreement between CACS PureCalcium and CACS TNC was detected, and in the in vivo patient study (N = 67), the accuracy of CACS quantification and classification was higher when using PureCalcium reconstructions in comparison to VNC reconstructions. It is important to underline that, as demonstrated both in vitro and in vivo, the accuracy of CACS quantification using VNC images is influenced by the keV level. Fink et al. demonstrated that the CACS for both the phantom and patient cases exhibited a notable rise as the keV levels decreased [85]. Mergen et al. showed in their study involving 90 patients that the best agreement between CACS quantified on VNC and TNC images was achieved at 70 keV [86].

### 5.3. Coronary Plaque Characterization

There is growing evidence that the consideration of the plaque type in addition to the plaque burden may enhance atherosclerosis imaging and risk prediction [87,88,89,90,91]. Plaque-type assessment can be divided into two approaches aimed at differentiating stable from unstable atherosclerosis and providing valuable insights into the vulnerability of plaques: anatomic assessment of plaque composition and molecular information about disease activity.

The anatomic assessment of plaque composition relies on the differentiation among calcified, fibrous, and lipid-rich plaques and the identification of features such as thinning of the fibrous cap or the presence of intraplaque hemorrhage. Conventional CT still has many limitations in accomplishing these tasks [92], and PCCT has proven useful in overcoming them. In an in vitro study, Rotzinger et al. simulated different patient sizes (small, medium, and large) and demonstrated, for all cases, the superiority of PCCT over conventional EID-CT in the detection of simulated non-calcified and lipid-rich plaques in coronary arteries [93]. In the study by Boussel et al., 23 plaques (10 calcified and 13 lipid-rich non-calcified) obtained from postmortem human coronary arteries were scanned with PCCT [8]. PCCT provided distinct visualization and differentiation of normal arterial wall, lipid-rich plaque, calcified areas, and surrounding adipose tissue based on their unique spectral attenuation characteristics and on the concentration of the iodine-based contrast agent used during imaging. Another ex vivo study demonstrated that PCCT was able to differentiate components such as calcium, iron, lipid surrogate, and cellular surrogate within the plaques by exploiting their distinct photoelectric and Compton effects [94]. Importantly, the correlation between PCCT findings and histological slices provided a valuable validation of the imaging technique.

Similarly, a recent study demonstrated no significant differences between PCCT-derived measurements and histological measurements of fibrous cap thickness, fibrous cap area, and lipid-rich necrotic core area [95]. Mergen et al. scanned 20 patients with atherosclerotic plaques in the proximal coronary arteries using PCCT and highlighted the capacity of ultra-high-resolution PCCT to provide enhanced and precise insights into atherosclerotic plaques [96]. Indeed, by reducing blooming artifacts, the ultra-high-resolution mode of PCCT heightened the depiction of non-calcified plaque elements (i.e., fibrotic and lipid-rich plaque components).

Molecular imaging techniques aim to provide information about the biological activity within the plaque, such as inflammation or neovascularization. These techniques typically involve the use of targeted imaging agents or molecular markers that can identify specific biological processes associated with plaque destabilization. Experiments conducted on phantom and animals illustrated the potential of utilizing PCCT with K-edge imaging in conjunction with gold nanoparticles as a compelling strategy for concurrently assessing lumen stenosis, plaque composition, vulnerability, and the detection of macrophages. In experiments conducted using phantoms and apo E-KO mouse models of atherosclerosis, Cormode et al. demonstrated that PCCT effectively discriminated among gold-based contrast agents, iodinated contrast agents, tissue, and calcium-rich matter [48]. This confirmed that PCCT could detect macrophages in cases of atherosclerosis while simultaneously visualizing vascular structures and calcified tissue. Si-Mohamed et al. performed imaging on atherosclerotic and control New Zealand white rabbits before and two days after injecting them with gold nanoparticles [21]. Their results showed that PCCT exhibited a stronger association between gold concentration and macrophage area compared to conventional CT (0.82 vs. 0.41). Additionally, only PCCT using gold K-edge imaging succeeded in distinguishing between the enhancement of the lumen (inner space) with an iodinated contrast material and the enhancement of the vessel wall using gold nanoparticles. This discrimination was confirmed by histology techniques such as transmission electron microscopy and inductively coupled plasma optical emission spectrometry.

### 5.4. Coronary Artery Stenting

Coronary artery stent implantation has become a widely accepted and effective nontreatment option for coronary artery stenosis to relieve symptoms, improve the patient’s quality of life, and decrease the risk of future cardiac events. In-stent restenosis (ISR) represents the main complication of this approach, occurring in 20–35% of bare-metal stents and only 5–10% of drug-eluting stents [97].

Coronary CT angiography demonstrated a high negative predictive value (98%) for ruling out significant ISR, emerging as a useful resource in the diagnostic workup of patients after coronary revascularization [98]. However, there is still a small percentage of stents that may not be accessible or accurately visualized due to metallic, blooming, and beam-hardening artifacts and the limited spatial resolution [99,100]. Several in vitro studies have evaluated the capability of PCCT to overcome these limitations. Mannil et al. imaged 18 coronary stents of different material compositions with both PC and conventional CT systems, maintaining identical imaging parameters [101]. PCCT provided better visualization of the stent lumen, reduced image noise and blooming artifacts, and improved overall image quality. In the study by Symons et al., superior visibility of the coronary stent lumen was demonstrated for high-resolution PCCT (voxel size of 0.25 mm) compared to standard-resolution PCCT (0.5 mm) and conventional dual-energy CT [102]. This finding is in agreement with the study of Petritsch et al., who scanned a body phantom stocked with 28 different coronary stents with PCCT operating in standard-resolution and ultra-high-resolution modes and conventional EID-CT system [103]. The PCCT in the ultra-high-resolution mode offered the best in-stent lumen visibility, while the PCCT in the standard resolution mode had the lowest noise levels

Furthermore, PCCT reconstructions at a standard voxel size (0.5 mm isotropic) had 25% less image noise than standard-resolution PCTT acquisitions. Von Spiczak et al. demonstrated that the application of a sharp convolution kernel tailored to the increased spatial resolution of PCDs enhanced the visualization of the coronary in-stent lumen [104]. Using iterative image reconstruction techniques considerably reduced the image noise caused by this approach. Rajagopal et al. demonstrated that, compared to EID systems, high-resolution PCCT reconstructions allowed for a more precise representation of high-contrast shape boundaries and lengths and for a reduction in metal blooming artifacts, which enabled a more accurate assessment of coronary lumen diameter [105].

Feuerlein et al. demonstrated the potential of PCCT in overcoming the limitations of conventional CCTA in a challenging scenario [106]. In their study, a phantom mimicking a low-density calcified plaque located within a coronary metal stent, with an attenuation level similar to that of the gadolinium-filled vascular lumen, was scanned with a PCCT system with six energy thresholds. Through the implementation of gadolinium K-edge imaging, a notable differentiation between the calcified plaque and the intravascular gadolinium and an effective mitigation of beam-hardening artifacts were achieved.

The advantages of PCCT in coronary stent evaluation have also been demonstrated by in vivo human studies. Boccalini et al. scanned eight patients with coronary stents with both PCCT and conventional CT. They demonstrated a reduced radiation dose and an increased objective and subjective stent and lumen visibility with fewer blooming artifacts for PCCT acquisitions [107]. Similarly, in the study by Si-Mohamed et al., all three radiologists who analyzed the patient images found that PCCT provided superior performance in terms of diagnostic quality for coronary stents than conventional CT, with an overall proportion of improvement of 92% [59].

In the context of the non-invasive detection of ISR, the relevant role of PCCT was demonstrated by an in vitro study wherein models simulating soft-plaque-like restenosis were inserted into ten different coronary stents positioned within plastic tubes filled with a contrast agent and were scanned by both PCCT and conventional CT [108]. Although both systems were able to effectively identify or raise suspicion about the presence of stenosis in all ten stents, PCCT enabled distinct and unambiguous visualization of the remaining lumen adjacent to the stenosis in seven stents. This level of visualization was not attainable using conventional CT.

**Figure 8:**
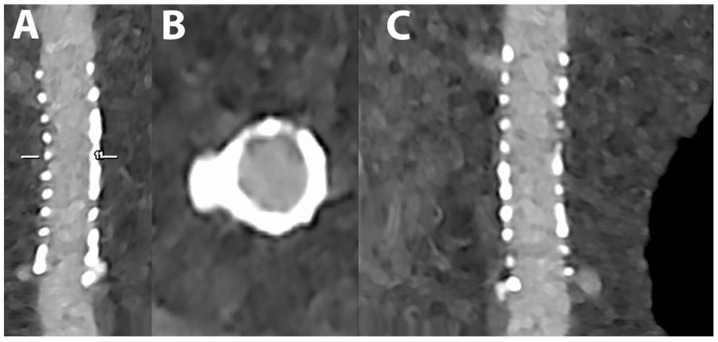
Cardiac/coronary PCCT example of coronary artery stent surrounded by calcification.

Figure 8 shows an imaging of coronary artery stent by PCCT.

The patient shows a stent in the proximal left anterior descending coronary artery (LAD) (A—reference stretched longitudinal MPR image; B—axial cross-section of proximal LAD; C—stretched longitudinal MPR with a higher reconstruction kernel).

The dataset was reconstructed using state-of-the-art photon-counting capabilities with ultra-high resolution achievable with PCCT. The scan was performed on a commercial whole-body Dual-Source Photon-Counting CT scanner (Naeotom Alpha, Siemens Healthineers) with 0.2 mm slice thickness (0.1 mm reconstruction increment and FOV 140 mm); the scan was performed with retrospective ECG gating with tube current modulation. In this case, the stent is perfectly patent without any sign of neointimal hyperplasia, and it is displayed with a resolution matrix of 1024 × 1024 pixels on the source axial reconstructions with a kernel filtering of B72 (vascular kernel sharp) and with maximum intensity of Quantum Iterative Reconstruction (QIR 4). The actual displayed resolution is 0.1 mm (100 microns).

The clarity and sharpness of the stent lumen and of the struts is unprecedented with previous CT scanners.

### 5.5. Myocardial Tissue Characterization

Myocardial diseases are characterized by alterations in tissue composition. These changes may involve the progression of myocardial fibrosis, the presence of edema, or the infiltration of fat, iron, or amyloid within the myocardium. Myocardial fibrosis is a common feature of various cardiac diseases and can be classified into two main types: interstitial fibrosis and replacement fibrosis.

Interstitial (diffuse) fibrosis is characterized by the diffuse spread of extracellular collagen without cardiomyocyte necrosis. Myocardial extracellular volume (ECV), a measure of the relative amount of extracellular matrix present in the entire myocardium, is used as a surrogate marker of interstitial fibrosis. Cardiac magnetic resonance (CMR) is currently considered the gold standard for non-invasive ECV quantification. However, recent studies have demonstrated that CT can serve as a valid alternative [109,110]. Two distinct methods are currently used to quantify ECV from CT data using conventional EIDs. The single-energy approach evaluates contrast media distribution and ECV by assessing the change in CT attenuation between the pre-contrast and late enhancement (LE) images acquired 3 to 5 min after contrast administration [111]. This method has been validated against histology and CMR [112,113]. The dual-energy acquisition only requires a single acquisition after contrast administration, and, therefore, it does not suffer from misregistration-related problems. This approach enables the generation of a VNC dataset, which provides information similar to what would be obtained from an actual non-contrast scan [114]. Mergen et al. demonstrated in vivo in 30 patients with severe aortic stenosis that the LE scan acquisition with PCCT was feasible at a low radiation dose [115]. The dual-energy- and single-energy-derived ECV measurements exhibited a high correlation (r = 0.87, *p* < 0.001) with a negligible mean error (0.9%). The strong correlation between the two approaches was confirmed by another in vivo study (r = 0.91, *p* < 0.001) involving 29 patients [116], where the dual-energy PCCT offered the advantage of a significantly lower (40%) radiation dose. Importantly, both dual-energy and single-energy PCCT techniques demonstrated a strong correlation and good reliability for ECV quantification compared to CMR.

Replacement (scar) fibrosis typically occurs due to myocardial necrosis and the subsequent loss of cardiomyocytes. Despite the use of dual-energy technology in CT, detecting myocardial scarring using LE methods is still limited by a relatively low CNR and an inferior ability to differentiate between healthy and fibrotic myocardium compared to CMR [117]. Symons et al. demonstrated in a canine model with myocardial infarction that PCCT was able to clearly discriminate the two injected contrast agents (gadolinium-based and iodine) [52]. Thanks to the integration of gadolinium, iodine, and soft-tissue maps, excellent contrast between infarcted myocardium, remote myocardium, and LV blood pool was achieved. Importantly, the accurate delineation of scar tissue by PCCT was confirmed by comparing PCCT findings with both CMR and histology analysis.

### 5.6. Myocardial Perfusion

Dual-energy CT-based iodine mapping has demonstrated high value in detecting myocardial perfusion defects during the first-pass myocardial perfusion assessment [118,119,120]. Indeed, the analysis of the iodine maps allows the detection of perfusion defects as areas with reduced or delayed iodine signal, indicating impaired blood flow to specific regions of the myocardium.

Phantom studies demonstrated the capability of PCCT to accurately quantify iodine concentrations across various levels and body sizes and to generate accurate CT numbers in virtual monochromatic images [36]. In a recent case report of a 61-year-old male with acute chest, dual-energy-derived iodine maps from PCCT revealed a reduced iodine concentration, suggestive of an inadequate blood supply, in the midventricular inferolateral wall [10]. CMR demonstrated a small ischemic transmural scar in the same area, confirming the findings from PCCT.

### 5.7. Myocardial Radiomics Features

Radiomics is a promising technique in medical imaging that aims to extract quantitative features from medical images, including textural information, which may not be readily apparent to the human observer [121]. The clinical implementation of radiomics in everyday clinical care is hindered by the challenge of feature stability. In particular, optimal spatial resolution and SNR play a critical role in extracting reliable and meaningful texture features [122]. Since PCCT can generate images with less noise, fewer artifacts, higher resolution, and improved CNR compared to conventional CT, it conveys the potential to improve radiomic analysis based on CT. Importantly, PCCT scans of organic phantoms demonstrated excellent test–retest stability for the vast majority of the radiomics features [123].

Ayx et al. compared in vivo the characteristics of radiomics features extracted from the human myocardium between PCCT and conventional CT [124]. While first-order radiomics features, describing basic statistical properties of the image intensity, were comparable between the two groups, higher-order radiomics features, capturing more complex patterns and relationships within the image, exhibited more heterogeneity and significant differences. Indeed, the enhanced resolution can lead to differences in the texture patterns captured by higher-order radiomics features. The same group demonstrated in another in vivo study (30 patients) that four radiomics-based texture parameters extracted from the myocardium were linked to the presence and extent of coronary artery calcification [125]. Moreover, the complexity of the texture features increased with increasing Agatston score, suggesting that as the calcification burden increases, the myocardial texture becomes more heterogeneous, exhibiting a greater variation in its structural patterns.

### 5.8. Epicardial and Pericoronary Adipose Tissue

Epicardial adipose tissue (EAT) represents the fat depot between the myocardium and the visceral layer of the pericardium. Pericoronary adipose tissue (PCAT) represents the adipose tissue within the EAT depot that encompasses the coronary arteries. These adipose tissues are not just passive energy storage sites but are also metabolically active and have been recognized as sources of various bioactive molecules and inflammatory mediators [126,127]. Several measurement methods have been reported in the literature for the evaluation of adipose tissue using CT. These methods include measuring the thickness, volume, and attenuation of the adipose tissue, with attenuation being the most commonly used parameter [128].

Mergen et al. assessed PCAT (right coronary artery) attenuation at different monoenergetic energy levels in 30 patients who underwent unenhanced and contrast-enhanced scans on a first-generation whole-body PCCT system [129]. The PCAT attenuation increased with increasing energy levels, and the presence of contrast material caused a small increase in PCAT attenuation, particularly at low energy levels.

Risch et al. demonstrated in vivo that the use of VNC reconstructions derived from PCCT datasets can be a viable alternative to TNC series for quantifying EAT volume [130]. Moreover, they tested different reconstruction methods, and the algorithm developed in the study showed superior and more consistent results for EAT volume when compared to TNC.

### 5.9. Reduction in Contrast Media Volume

The improved CNR achieved through PCCT offers the possibility of reducing the required amount of iodine-based contrast media. The fact that the rate of attenuation by iodine rises as X-ray energy decreases enables to administer a reduced quantity of intravenous contrast agent while achieving diagnostic outcomes comparable to those obtained from a conventional CT scan administered at a full dose.

Emrich et al. demonstrated in a dynamic coronary artery phantom that the use of virtual monoenergetic image reconstruction at 40 KeV on PCCT allowed to achieve a 50% reduction in contrast media concentration while still achieving the necessary diagnostic attenuation and maintaining the desired objective image quality for coronary CT angiography [131].

Cundari et al. proved in their in vivo human study that the advantageous properties of low-energy-level (45 keV) VMI reconstructions through PCCT made it possible to decrease the contrast media volume by 40% in coronary computed tomography angiography without compromising the diagnostic image quality [132].

## 6. Conclusions

Non-invasive cardiovascular imaging has seen significant advancements in the past decades, and the introduction of PCDs is a notable development in this field. PCCT offers several advantages over traditional CT technologies, including improved spatial resolution, improved signal and contrast behavior, reduced electronic noise, decreased radiation exposure, and multi-energy capability. These advantages have demonstrated promising benefits in the qualitative assessment of coronary plaques and stents and in the quantification of coronary calcium, myocardial extracellular volume, myocardial radiomics features, and epicardial and pericoronary adipose tissue.

Continued research, technological advancements, and clinical implementation will further uncover the full potential of PCDs and solidify their role in enhancing diagnostics, improving risk stratification, and optimizing treatment strategies in cardiovascular diseases.

## Figures and Tables

**Table 1:** Cardiovascular applications of PPCT.

Cardiovascular Applications of PCCT
Improved visualization of coronary plaques and patent lumen over conventional CT
Superior accuracy in the quantification of luminal stenosis across all plaque types compared to conventional CT
Improved accuracy in coronary artery calcium quantification compared to conventional CT
Improved detection of coronary calcium even at a reduced radiation dose compared to conventional CT
Anatomic assessment of plaque composition: differentiation among calcified, fibrous, and lipid-rich plaques and identification of features such as thinning of the fibrous cap or presence of intraplaque hemorrhage
Potential capability to provide information about the biological activity within the coronary plaque, such as inflammation or neovascularization
Better visualization of the stent lumen compared to conventional CT
Improved detection of in-stent restenosis compared to conventional CT
Quantification of myocardial extracellular volume at a low radiation dose
Accurate delineation of myocardial scar achieved thanks to the excellent contrast between infarcted myocardium, remote myocardium, and left ventricular blood pool
Detection of myocardial perfusion defects
Improved extraction myocardial radiomics features compared to conventional CT
Accurate quantification of epicardial adipose tissue volume and assessment of pericoronary adipose tissue attenuation
Reduction in the volume of iodine-based contrast media in coronary CT angiography without compromising the diagnostic image quality

## Data Availability

Not applicable.
